# Global and national influenza-associated hospitalisation rates: Estimates for 40 countries and administrative regions

**DOI:** 10.7189/jogh.13.04003

**Published:** 2023-01-27

**Authors:** John Paget, Lisa Staadegaard, Xin Wang, You Li, Tayma van Pomeren, Jojanneke van Summeren, Michel Dückers, Sandra S Chaves, Emily K Johnson, Cédric Mahé, Harish Nair, Cecile Viboud, Peter Spreeuwenberg

**Affiliations:** 1 Netherlands Institute for Health Services Research (Nivel), Utrecht, the Netherlands; 2 School of Public Health, Nanjing Medical University, Nanjing, China; 3 Centre for Global Health, Usher Institute, University of Edinburgh, Edinburgh, UK; 4 Foundation for Influenza Epidemiology, Fondation de France, Paris, France; 5 Institute of Health Metrics and Evaluation, University of Washington, Seattle, USA; 6 Fogarty International Center, National Institutes of Health, Bethesda, USA

## Abstract

**Background:**

WHO estimates that seasonal influenza epidemics result in three to five million cases of severe illness (hospitalisations) every year. We aimed to improve the understanding of influenza-associated hospitalisation estimates at a national and global level.

**Methods:**

We performed a systematic literature review of English- and Chinese-language studies published between 1995 and 2020 estimating influenza-associated hospitalisation. We included a total of 127 studies (seven in Chinese) in the meta-analysis and analyzed their data using a logit-logistic regression model to understand the influence of five study factors and produce national and global estimates by age groups. The five study factors assessed were: 1) the method used to calculate the influenza-associated hospitalisation estimates (rate- or time series regression-based), 2) the outcome measure (divided into three envelopes: narrow, medium, or wide), 3) whether every case was laboratory-confirmed or not, 4) whether the estimates were national or sub-national, 5) whether the rates were based on a single year or multiple years.

**Results:**

The overall pooled influenza-associated hospitalisation rate was 40.5 (95% confidence interval (CI) = 24.3-67.4) per 100 000 persons, with rates varying substantially by age: 224.0 (95% CI = 118.8-420.0) in children aged 0-4 years and 96.8 (95% CI = 57.0-164.3) in the elderly aged >65 years. The overall pooled hospitalisation rates varied by calculation method; for all ages, the rates were significantly higher when they were based on rate-based methods or calculated on a single season and significantly lower when cases were laboratory-confirmed. The national hospitalisation rates (all ages) varied considerably, ranging from 11.7 (95% CI = 3.8-36.3) per 100 000 in New Zealand to 122.1 (95% CI = 41.5-358.4) per 100 000 in India (all age estimates).

**Conclusions:**

Using the pooled global influenza-associated hospitalisation rate, we estimate that seasonal influenza epidemics result in 3.2 million cases of severe illness (hospitalisations) per annum. More extensive analyses are required to assess the influence of other factors on the estimates (e.g. vaccination and dominant virus (sub)types) and efforts to harmonize the methods should be encouraged. Our study highlights the high rates of influenza-associated hospitalisations in children aged 0-4 years and the elderly aged 65+ years.

Influenza is a common respiratory disease with high incidence rates reported in countries worldwide [1,2]. Despite its substantial health and economic impact, information regarding influenza and its associated severity, including hospitalisations, is still limited [3,4]. Understanding the burden of severe illness by age group is particularly relevant during peak viral transmission and is important for informing decisions regarding vaccination target groups. The World Health Organization (WHO) highlights the importance of vaccinating pregnant women, young children (six months to five years), the elderly (aged more than 65 years), individuals with chronic medical conditions, and health care workers [1].

WHO estimates that annual influenza epidemics result in roughly three to five million cases of severe illness (hospitalisations [5]) annually [1]. There are many different approaches for assessing the burden of severe influenza cases, ranging from the use of International Statistical Classification of Diseases and Related Health Problems (ICD) codes to identify hospitalised cases [2], population-based sentinel surveillance networks based on laboratory-confirmed influenza hospitalisations [6,7], literature reviews and modeling procedures to summarize the findings [8] and the use of time series regression models reporting excess hospitalisations [3]. Efforts to estimate the burden of influenza disease at a national level have increased in recent years, especially following the publication of the WHO manual on estimating the disease burden associated with seasonal influenza [7]. Most of these studies have been performed at a local, national or regional (eg, a WHO region) level, but two recent studies have made global all-age estimates of 3.7-22.9 million hospitalisations due to influenza-related lower respiratory infections [9] and 4.0-8.7 million influenza-associated respiratory hospitalisations [10].

A recent literature review of influenza-associated hospitalisation rates (from 2007 to 2018) identified variability in the methods, case definitions, and data sources, concluding that calculating a pooled estimate was impossible due to extremely high heterogeneity in estimates observed across studies [3]. While we agree with this study regarding the challenges of a pooled analysis, we aimed to assess the heterogeneity of the estimates and how they could be potentially pooled. We did this by first updating the literature review (until 2020) and then assessing the role of five study factors on the hospitalisation estimates, with the study factors based on data uniformly available from all studies. Our second objective was to produce pooled national and global estimates of influenza-associated hospitalisations by age groups while considering these five study factors. This analysis provides a more up-to-date literature review, assesses the influence of study design factors on the influenza hospitalisation burden estimates, and produces hospitalisation estimates for 40 countries and administrative regions for three different age groups (0-4 years, >65-year-olds, and all ages).

## METHODS

### Literature search

We searched MEDLINE, Embase, CNKI, Wanfang, and Chongqing VIP (with the last three being Chinese databases) for English- and Chinese-language studies, following the Preferred Reporting Items for Systematic Reviews and Meta-Analyses (PRISMA) guidelines. The search included a combination of search terms related to 1) hospitalisation, 2) influenza and 3) “influenza associated” or “excess”. Details of the literature search can be found in the **Online Supplementary Document**.

### Study selection

We included studies that were published between 1995 and the end of March 2020, that reported hospitalisation rates for the general population, and that focused on total influenza (2009 pandemic estimates were excluded). We pooled estimates (where possible) with an influenza (sub)type focus. Studies from all countries and/or regions were eligible and there was no restriction by age groups (for the literature review). Two types of studies were eligible for inclusion in the literature review: rate-based studies and time series regression-based studies. Rate-based studies were defined as studies that report age group-specific hospitalisation rates for influenza using primary data (generally based on laboratory confirmation or ICD-coded diagnosis) and applying these to a population denominator, ie, catchment population. Time series regression model studies were defined as studies that estimate excess hospitalisations (using regression methods). A full and detailed description of the literature review can be found in the **Online Supplementary Document**.

### Study design characteristics

When extracting the data, we characterized the studies based on five factors that are readily available in all papers: 1) method (rate- or time series regression-based), 2) outcome measure (divided into three envelopes: narrow, medium, or wide), 3) laboratory-confirmed or not, 4) national or sub-national data, and 5) if the rates were based on a single year or multiple years. The “narrow” envelope was based on laboratory confirmation and a clinical diagnosis of influenza, the “medium” envelope was based on broad definitions of respiratory syndromes (e.g. influenza-like illness, acute respiratory illness) without laboratory confirmation, and the “wide” envelope was defined as broader disease categories that can be associated with influenza (e.g. circulatory and respiratory diseases, chronic obstructive pulmonary disease), without laboratory confirmation (see the **Online Supplementary Document** for definitions).

### Statistical analyses

We used a multilevel (meta-analysis) logit-logistic regression model to generate national and global estimates of influenza-associated hospitalisations [11,12]. The outcome measure (dependent variable) was the influenza-associated hospitalisation rate per 100 000 population per season (or average over several seasons). The model (**Online Supplementary Document**) consisted of four levels, with level 1 being the individual estimates per study; at this level, binomial error variance (constrained to 1) was modeled. Level 2 modeled the variance between individual measurements within studies, level 3 modeled between-study variance, and level 4 (cross-classified) modeled between-country variance. The fixed estimate of the model was the overall average rate (similar to the overall rate in other forms of meta-analysis). The five study design factors were also modeled, as they could influence the estimates. Every study factor was given an equal weight (indicator scoring (0,1) minus 1/N(cat), N(cat) = number of categories in factor). Additionally, we added control (or adjustment) factors if an outcome did not follow the exact age limits (e.g. if the outcome was reported for participants aged 60 years and older).

We estimated all models using restricted iterative generalized least squares (RIGLS) with first-order penalised quasi-likelihood (PQL). The overall pooled hospitalisation rate was equally weighted for the five factors and for categories within a factor. No weight was added for differences in sample size, as is very often done in meta-analyses, primarily because the heterogeneity between individual measures and studies was so large that weighting for sample size would make the different model estimates significantly more difficult to interpret.

We calculated country estimates using an empirical-Bayes procedure in the multilevel models. Notably, a country estimate can differ strongly from the published country rate as the country estimates are adjusted for the five factors included in the model, like the overall pooled estimated rates. For every age category (0-4, >65, and all age) a separate model was estimated.

## RESULTS

### Study selection

**Figure 1:**
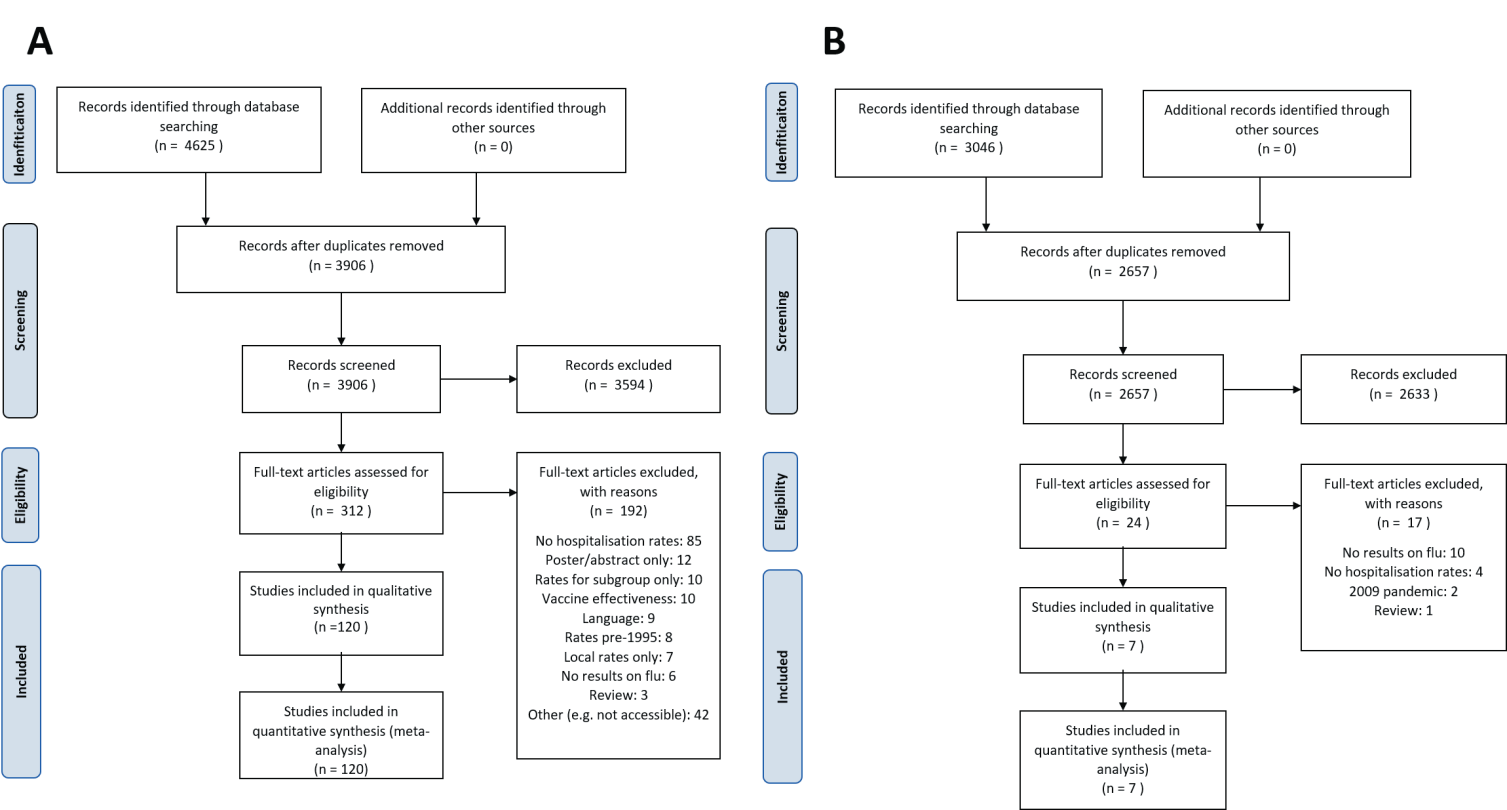
Systematic literature review PRISMA flowchart. **Panel A:** English language literature. **Panel B:** Chinese language literature.

The English literature search retrieved a total of 4625 records. After deduplication, 3906 records remained for title and abstract screening, after which 312 records remained for full-text review. A total of 120 studies were included for data extraction. The Chinese literature search retrieved 3046 records. After deduplication, 2657 records were screened by title and abstract, 24 of which proceeded to the full-text screening. A total of seven studies were included for data extraction (**Figure 1**). Finally, we included 127 papers that reported influenza-associated hospitalisations (**Table 1** and Table S1 in the **Online Supplementary Document**). Considering two papers reported multiple country estimates [13,14], we had a total of 132 country estimates stemming from 46 different countries/administrative regions.

**Table 1:** Characteristics and countries included in the literature review and the final analysis

WHO region (n of countries)		Countries		Number of estimates in literature review		Number of studies in final analysis		Factor 1: type of study				Factor 2: measurement outcome (envelope)*					Factor 3: laboratory-confirmed				Factor 4: national or regional estimate				Factor 5: single- or multiple-season estimates				Number of influenza hospitalisation estimates included in the analysis		Seasons (range)		Percentage of world population (2013)†	
								**Rate-based**	**Time series regression-based**			**Narrow**		**Medium**		**Wide**	**Yes**		**No**		**National**		**Regional**		**Single**		**Multiple**							
**Sub-Saharan Africa (AFRO) (n = 8)**		D.R. Congo		1		1		1				1					1				1						1		4		2013-2015		1.0%	
		Ghana		1		1		1				1					1						1				1		3		2013-2015		0.4%	
		Kenya		5		5		5				3		3			3		3		2		3		3		4		21		2007-2014		0.6%	
		Madagascar		1		1		1				1					1				1				1		1		15		2011-2016		0.3%	
		Rwanda		1		1		1				1					1				1						1		2		2012-2014		0.2%	
		South Africa		1		1			1					1		1			1		1						1		2		2007-2012		0.7%	
		Uganda		1		1		1				1					1						1				1		16		2013-2016		0.5%	
		Zambia		1		1		1				1							1		1						1		8		2011-2014		0.2%	
																	
**North Africa and Middle East (EMRO) (n = 2)**		Egypt		1		1		1				1					1						1		1				1		2013		1.2%	
		Oman		2		2		2				1		1			1		1		1		1		2		1		48		2008-2015		0.1%	
**Europe (EURO) (n = 12)**		Austria		1																														
		Finland		1		1		1				1					1				1						1		3		1996-2009		0.1%	
		France		1		1		1				1							1		1				1		1		6		2012-2017		0.9%	
		Germany		1																														
		Greece		1																														
		Netherlands		1		1			1					1		1			1		1						1		3		1997-2003		0.2%	
		Norway		1		1		1				1							1		1				1		1		10		2008-2017		0.1%	
		Portugal		1		1			1					1					1		1				1		1		28		1998-2015		0.1%	
		Romania		1		1		1						1					1		1				1				5		2011-2016		0.3%	
		Spain		4		3		3				3					3				2		1				3		13		1999-2016		0.7%	
		Switzerland		1																														
		United Kingdom		4		4		1	3			2		1		1	1		3		1		3		1		3		8		1996-2009		0.9%	
**Americas (PAHO) (n = 10)**		Argentina		2		1			1					1		1			1		1				1				8		2005-2008		0.6%	
		Bolivia		1		1		1				1		1			1		1				1		1		1		26		2012-2017		0.2%	
		Canada		6		3		1	2			2		2			1		2		1		2		1		2		6		1998-2014		0.5%	
		Chile		1		1		1						1					1		1				1		1		8		2012-2014		0.2%	
		Costa Rica		2		2		2				1		1			1		1		2				2				17		2009-2012		0.1%	
		El Salvador		1		1		1						1					1		1				1				8		2009-2012		0.1%	
		Guatemala		2		2		2				1		1			1		1		1		1		2		1		36		2009-2012		0.2%	
		Honduras		1		1		1						1					1		1				1				8		2009-2012		0.1%	
		Nicaragua		1		1		1						1					1		1				1				8		2009-2012		0.1%	
		United States		36		19		14	5			14		3		4	14		5		12		7		16		6		157		1979-2019		4.4%	
**South-East Asia (SEARO) (n = 5)**		Bangladesh		2		2		2				2					2				2				2		1		9		2008-2014		2.2%	
		Bhutan		1		1		1				1				1	1		1		1				1				17		2015-2016		0.0%	
		India		2		2		2				2					2						2		2				16		2009-2012		17.9%	
		Indonesia		1		1		1				1					1						1		1		1		7		2013-2016		3.5%	
		Thailand		1																														
**Western Pacific (WPRO) (n = 9)**		Australia		4		4		1	3			1		3		2			4		2		2				4		9		1996-2009		0.3%	
		Cambodia		2		2		2				1		2			1		2		1		2		2				15		2015-2016		0.2%	
		China – Mainland		12		10		8	2			8		4			8		4				10		10		4		54		2005-2018		19.0%	
		China – Hong Kong		9		7		1	6			1		4		4	1		6				7		3		4		35		1996-2013		0.1%	
		New Zealand		4		3		2	1			2		1		1			3		2		1		1		2		5		1994-2008		0.1%	
		Singapore		3		2			2					2					2		2				1		2		29		2004-2014		0.1%	
		South Korea		1		1		1						1					1		1				1				12		2002-2005		0.7%	
		Taiwan		1																														
		Vietnam		2		1		1				1		1			1		1				1		1		1		24		2009-2013		1.3%	
**Total**		46		131		96		68	28			58		40		16	50		53		49		48		64		53		710		1979-2019		60.2%	
**Total (%)**	-		-		-		29%			51%	35%		14%		49%					51%		49%		55%		45%		-		-		-	

WHO – World Health Organization, AFRO – Regional Office for Africa, EMRO – Regional Office for the Eastern Mediterranean, EURO – Regional Office for Europe, PAHO – Pan-American Health Organization, SEARO – Regional Office for South East Asia, WPRO – Regional Office for Western Pacific

*A detailed outline of the definitions used can be found in the **Online Supplementary Document**. A “narrow” envelope was defined as laboratory-confirmed influenza and a clinical diagnosis of influenza (ICD code influenza in primary position). A “medium” envelope was defined as any of the following disease outcomes without a (systematic) laboratory test confirmation: acute respiratory infection (ARI), influenza-like illness (ILI), acute lower respiratory tract infection (ARLI), severe acute respiratory infection (SARI), lower respiratory tract infection (LRTI), upper respiratory tract infection (URTI), pneumonia & influenza (P&I). A “wide” envelope was defined as any of the following disease outcomes without a (systematic) laboratory test confirmation: circulatory and respiratory diseases, chronic obstructive pulmonary disease (COPD), stroke, critical illness, congestive heart failure, LRTI & pulmonary diseases.

†World Population Data Set, 2013 (https://www.prb.org/wp-content/uploads/2015/01/2013-population-data-sheet_eng.pdf).

### Description of the included studies

When incorporating these 132 country estimates into our data extraction template, we could only use 96 estimates (from 91 papers), with most estimates being rejected due to not fitting the selected age groups or having no information available on 95% confidence intervals (CIs) or standard errors (SEs). The 96 estimates came from a total of 40 countries/administrative regions (**Table 1**), ranging from two countries in North Africa and the Middle East (the WHO Eastern Mediterranean region) to 10 in the Americas. Importantly, there were country estimates from all six WHO regions. Regarding estimates per country, the United States (n = 19) and China (n = 17) (including Hong Kong (n = 7)) were particularly well represented, providing 36 (38%) of the total estimates.

Among the estimates included in the analysis (**Table 1**), rate-based studies represented 71% of the estimates and time series regression-based studies represented 29% (Factor 1). Importantly, the rate-based studies were well represented across all WHO regions, while the time series regression-based studies were mainly carried out in high-income countries. There were similar numbers of national (51%) and subnational estimates (49%). For the outcome measure (Factor 2), there was a predominance of “narrow” and “medium” envelopes (representing 86% of all estimates). Laboratory confirmation (Factor 3), national vs subnational (Factor 4), and single-year estimates (Factor 5) were reported in roughly half of the studies. The seasons covered by the studies were from 1979-2019, while high-income countries had longer time series data compared to low- or middle-income countries (where data was typically post-2009). Papers typically included multiple estimates per country (multiple seasons or multiple age groups), so we were able to extract a total of 710 hospitalisation estimates for the modeling procedure, with 22% from the US and 13% from China (including Hong Kong).

### Influence of the five factors on the hospitalisation rates

The results from the multi-level modeling analyses are presented in **Table 2**. For the fixed effects analysis, the national estimates were higher than the subnational estimates in all age groups (all not significant, Table S2 in the **Online Supplementary Document**). We also found that the rate-based estimates were higher than the time series regression-based estimates in all age groups (significant for all ages and children aged 0-4, Table S2 in the **Online Supplementary Document**). For the influence of the envelope and laboratory testing, we found that the wider the envelope, the higher the estimate (although the differences were only significant for all ages) and studies that included a laboratory test had lower rates in all age groups (Table S2 in the **Online Supplementary Document**). Studies that were based on multiple years tended to have lower rates than single years (only significant in all ages, see Table S2 in the **Online Supplementary Document**).

**Table 2:** Pooled influenza hospitalisations fixed and random effects estimates

Factors	All ages		Children aged 0-4 y		Elderly (>65 y)	
	**Average hospitalisation rate per 100 000**	**95% CI**	**Average hospitalisation rate per 100 000**	**95% CI**	**Average hospitalisation rate per 100 000**	**95% CI**
**Overall estimate**	40.5	24.3-67.4	224.0	118.8-420.0	96.8	57.0-164.3
**Fixed effects: independent factors**						
						
						
						
Factor 1:						
*Rate-based studies*	67.3	39.1-118.0	454.2	263.9-780.8	97.6	54.7-174.1
*Time series regression-based studies*	24.1	10.9-53.2	110.3	42.9-283.1	95.9	41.6-220.9
Factor 2:						
*Narrow envelope*	39.1	21.1-72.6	162.9	83.7-316.9	11.6	58.2-213.8
*Medium envelope*	28.2	14.5-54.8	190.5	79.9-453.5	60.6	31.9-115.1
*Wide envelope*	60.3	30.3-120.0	361.9	140.5-929.2	133.9	50.9-351.4
Factor 3:						
*Laboratory test – yes*	17.2	7.8-37.9	110.8	40.3-303.7	32.0	14.4-71.4
*Laboratory test – no*	95.4	56.3-161.5	452.3	250.2-816.5	292.1	162.9-523.1
Factor 4:						
*National*	52.7	27.5-100.9	266.4	135.9-521.6	112.6	60.1-211.0
*Sub-national*	31.1	17.9-54.0	188.3	89.1-397.1	83.2	43.9-157.6
Factor 5:						
*Multiple years of data*	30.7	17.5-53.6	207.3	108.9-394.3	105.6	58.3-191.2
*One year of data*	53.5	30.8-93.0	241.9	124.7-468.8	88.7	48.8-161.0
**Random effects: three levels**						
Measurement outcome (Level 2)	40.5	11.2-146.8*	224.0	61.1-817.2*	96.8	26.7-349.8*
Study (Level 3)	40.5	5.7-286.3*	224.0	40.8-1220.3*	96.8	16.9-551.4*
Country (Level 4)	40.5	8.6-190.1*	224.0	57.0-875.1*	96.8	21.9-427.5*

CI – confidence interval

*95% range interval = 95% of the individual rates at this level fall within this interval (this is corrected for other levels and the fixed effects).

For the random effects analysis, we found that variance was mostly observed at the study level (between study variance) (Table S2 in the **Online Supplementary Document**), followed by the country level (between country variance) and the measurement outcome level (between individual estimates per study).

### Pooled influenza-associated hospitalisation rates

**Figure 2:**
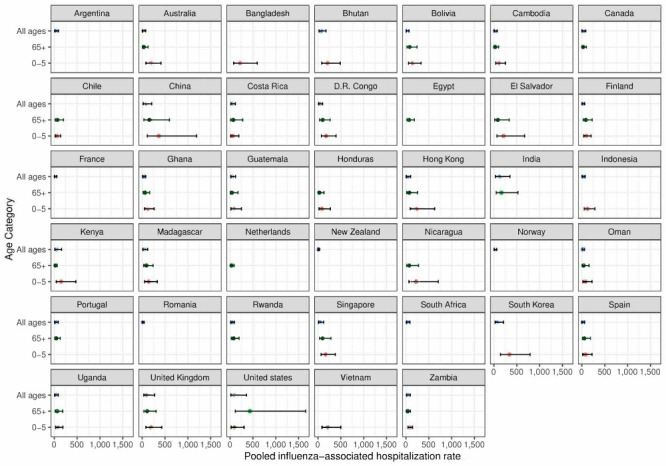
Pooled influenza-associated hospitalisation rates by included country and age group.

**Figure 3:**
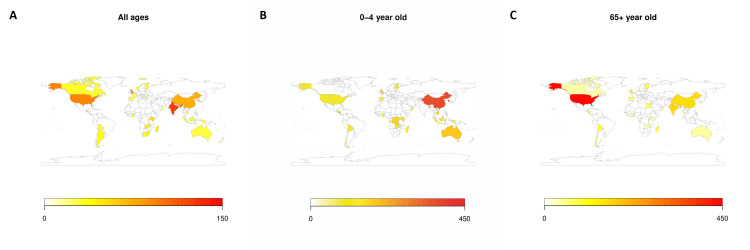
Pooled hospitalisation rates by included country and age group. **Panel A:** Countries – all ages. **Panel B:** Countries – children aged 0-4. **Panel C:** Countries – elderly, 65 years and older.

The calculated pooled global influenza-associated hospitalisation rate was 40.5 (95% CI = 24.3-67.4) per 100 000 persons, with rates varying substantially by age: 224.0 (95% CI = 118.8-420.0) in children aged 0-4 and 96.8 (95% CI = 57.0-164.3) in the elderly aged >65 (**Table 2**). We also calculated national hospitalisation rates for all countries where data were available (**Figure 2**), with the estimates presented on a world map in **Figure 3** and the precise country estimates presented in Table S3 in the **Online Supplementary Document****.** **Figure 1** shows that the hospitalisation rates were highest in the 0-4 age group in all countries except the US, where the highest rate is in the >65 age group. This is mainly due to the extreme variability in published US estimates: several studies, but not all, reported extremely high rates for the elderly. The country hospitalisation rates (all ages) varied widely worldwide, ranging from 122.1 (95% CI = 41.5-358.4) per 100 000 in India and 92.6 (95% CI = 23.5-364.7) in the United States to 11.7 (95% CI = 3.8-36.3) in New Zealand and 18.6 (95% CI = 6.1-56.6) in Oman. Importantly, influenza hospitalisation rates were higher in mainland China than in Hong Kong in all age groups.

## DISCUSSION

We used 710 published influenza-associated hospitalisation estimates from around the world to calculate global and 40 national influenza-associated hospitalisation rates for all ages, children aged 0-4 years, and the elderly aged 65 years or older. Our analysis of the five extracted study factors that influence these rates indicates that the rate-based estimates tended to be higher (vs time series regression-based estimates), laboratory-confirmed studies tended to have lower rates (vs non-laboratory confirmed studies) and a broader outcome measure had higher rates. An important advantage of applying our modeling approach is that the country and global estimates are more comparable, as the method assumes they were calculated with the same (five) study characteristics. Using this approach, the national hospitalisation rates varied widely, ranging from 11.7 (95% CI = 3.8-36.3) per 100 000 in New Zealand to 122.1 (95% CI = 41.5-358.4) per 100 000 in India (all age estimates). The global pooled influenza-associated hospitalisation rate using all studies was 40.5 (95% CI = 24.3-67.4) per 100 000 population, with rates varying substantially by age: 224.0 (95% CI = 118.8-420.0) per 100 000 in children (aged 0-4) and 96.8 (95% CI = 57.0-164.3) in the elderly (>65).

Our study used a similar design to a previous study which investigated influenza-associated hospitalisation rates worldwide [3]. However, we advanced the analysis by using a multilevel (meta-analysis) logit-logistic regression model to adjust for five different study factors and calculate pooled national and global estimates. The former will help researchers understand the influence of various factors on influenza-associated hospitalisation estimates and improve the interpretation of future studies.

Notably, our choice of the five factors was based on what could be directly extracted from the 127 papers identified in our literature review, which was reflected in the high levels of unexplained heterogeneity in our models. In the future, other factors should be considered for this type of analysis, including: vaccination coverage rates, co-morbidities, the underlying population distributions (e.g. percentage of the population aged under 15 years [15]), socio-demographic factors (e.g. the Socio-Demographic Index [16]), the circulating viruses [16], hospitalisation polices (e.g. government subsidised hospitalisations for certain age groups which affects their representation in the data), the impact of ICD coding on the estimates (inclusion of primary, secondary, and any ICD codes [2]), and even societal and cultural factors which affect health care usage. These different factors could significantly impact hospitalisation rates and, therefore, should be considered when pooling data from diverse settings.

This study can be seen as a pilot study, as it only focuses on the influence of five factors on influenza-associated hospitalisation rates. A larger study should be performed which extends the analysis to a wider range of factors (e.g. vaccination coverage rates, co-morbidities, hospitalisation polices, etc.), as this would allow for a better definition and understanding of the heterogeneity of the rates and for improving the pooled estimates. Ideally, researchers would use this approach to make national, regional, and global estimates, requiring sufficient national estimates from each world region. Importantly, our study highlights the need for high quality, and harmonized (eg, data sources, calculation methods, and reporting of results) estimates of influenza-associated hospitalisation rates, as these are important for research and policy means.

We gathered the influenza-associated hospitalisation rates based on two general approaches: rate-based estimates and time series regression-based estimates. These two methods are inherently different, as the first is typically based on hospital data (with cases often laboratory-confirmed) and the second is a modeling exercise (using various linear regression methods [3]) where excess hospitalisations are calculated based on hospitalisation data for a certain outcome (e.g. respiratory hospitalisations) together with virologic surveillance data. Importantly, our analysis suggests that the rate-based estimates tended to be (on average and considering the four other study factors) higher than the time series regression-based estimates and the effect differed by age (it was stronger in the all age category and the elderly). These results were not entirely unexpected as Roguski et al. [3] did not find clear differences between the two sets of estimates (the regression estimates were more tightly grouped).

A striking finding of our analysis is that, while the US had an influenza-associated hospitalisation age signature that had the highest rates in the elderly (>65), the highest rates in all other countries, after adjusting for the five factors, were in children aged 0-4 years, in both high- and low-income countries (**Figure 2**). This is mainly due to the extreme variability in the US-published elderly estimates, with several studies, but not all, reporting extremely high rates for this age group. Other factors that explain this finding are the age signature of hospitalisation rates in low-income countries [3] and China (which provided many estimates), and each data point (country estimate) having an equal weight in our model.

Another Burden of Influenza and RSV Disease (BIRD) study modeled the impact of influenza and respiratory syntactical virus (RSV) on hospitalisation admission rates based on ICD codes for acute respiratory infections and the proportion of cases that were RSV- and influenza-positive (laboratory-confirmed) [2]. By modeling the rates by age group, the study found that the highest influenza-associated hospitalisations were in children aged 0-4 (especially in the 0-2 age group, a sub-analysis that we were unable to perform) [2]. Importantly, the study (which focused on the primary ICD codes) found only slightly higher hospitalisation rates for the elderly than for the adults and a similar age influenza-associated hospitalisation signature using a very different approach (based on administrative data).

Our pooled estimates of influenza-associated hospitalisations can be used as a reference for comparison purposes, either at a national (e.g. in the US [17,18]) or at a global level. At a global level, they can be compared to other estimates of influenza-associated hospitalisations (**Table 3**). The methods used to make these estimates differ widely, with some based on a meta-analysis of published and un-published estimates in two age groups (Global Respiratory Hospitalisations-Influenza Proportion Positive (GRIPP) estimates) [19,20] and others that use various modeling methods (imputations for the IceBerg group [10] and a counterfactual approach for the Global Burden of Disease Study [9]). Three studies (**Table 3**) have reported a global estimate for all ages, and these range from 40.5 per 100 000 (our estimate) to 123.8 per 100 000 (the Global Burden of Disease (GBD) estimates [9]). Regarding the total number of hospitalisations, the estimates vary widely from 3.2 million for the BIRD project to 9.46 million estimated by GBD (**Table 3**) [9]. There is some difficulty in comparing the estimates from the different groups (e.g. not all metrics are available), but we do find that the BIRD estimates are lower than the other groups (**Table 3**) and more in line with the CDC IceBERG project (3.95-8.72 million cases per annum) and the WHO estimate of three to five million annual cases of severe illness [1,5]. It will be important to perform more detailed comparisons of these different estimates, so that an overall estimate (an “ensemble” estimate) can be made, as has been done by WHO for influenza mortality [21].

**Table 3:** World influenza-associated hospitalisation estimates: BIRD, IceBerg, IHME, and GRIPP literature reviews*

Research group	Details	All age rate (95% CI)	0-4 age rate (95% CI)	Age >65 rate (95% CI)	World estimate (95% CI)	% 0-4	% 65+
**BIRD influenza estimates**	Literature review (1995-2020), multi-level model with three levels: outcome envelope, type of study and country.	40.5 (24.3-67.4)	224.0 (118.8-420.0)	96.8 (57.0-164.3)	3.18 million (1.91-5.28 million)	48%	23%
**IceBerg project [** 10 **]**	Literature review, extrapolation of national estimates using an imputation procedure.	51.8-114.5	201.2-488.7	123.4-549.4	3.95-8.72 million	37%	28%
**GBD study [** 9 **]**	Counterfactual approach that estimated the LRTI incidence, hospitalisations, and mortality. A fraction was then attributed to each outcome for influenza. 2017 GBD estimate [9].	123.8 (48.5-300.2)	NA	NA	9.46 million (3.71-22.94 million)	NA	NA
**GRIPP literature reviews**	Influenza-associated lower respiratory tract infections and hospitalisations. Systematic literature review and meta-analysis. Children (1982-2012) [19] and adults (2016) [20].	Children (0-4): 135 (95-193), adults (≥20 y): 115 (65-190)	Children (0-4): 135 (95-193)	Elderly (>65 y): 437 (265-612)	Children: 870 000 (610 000-1.24 million), adults: 5. 68 million (3.21-9.43 million)	13%	43%

CI – confidence interval, y – years, NA – not applicable, BIRD – Burden of Influenza and RSV Disease, GRIPP – Global Respiratory Hospitalisations-Influenza Proportion Positive, IHME – Institute for Health Metrics Evaluation, GBD – Global Burden of Disease, LRTI – lower respiratory tract infection

*All estimates were directly extracted from the manuscripts/presentation or, when only a rate was available, are based on UN world population estimates for 2020 (United Nations, Department of Economic and Social Affairs, Population Division (2022). Data Portal, custom data acquired via website. United Nations: New York. Available from https://population.un.org/DataPortal/ (Accessed: 31 December 2022).

Our study has several strengths and limitations. Two important strengths are that we also reviewed the Chinese-language literature (3046 papers based on our inclusion criteria) and our modeling approach allows one to assess the influence of five factors on the hospitalisation estimates and to calculate pooled estimates. Our literature review was also more extensive than the previous one [3], as it covered a longer time period: 1995-2020 compared to 2007-2018, which is likely the reason why we included more studies (130 vs 98) and why the proportion of time series regression-based excess estimates (which have become more popular over time) was higher (29% vs 19% [17]). One limitation of our study is that we only included scientific papers in our analysis and did not extend our assessment to the unpublished or gray literature. Another limitation is that we had to exclude many studies/estimates, as we limited our analysis to studies that met our age groups or included CIs or SEs (the total number of estimates declined from 2670 to 711). Finally, we did not weight the studies included in our analysis (e.g. based on the size or a quality assessment of the study) or analyze the influenza-associated hospitalisation rate for the subgroup of adults aged 5-64 age group, which limits our overall assessment of the burden in different age groups.

## CONCLUSIONS

Our study highlights systematic variation in influenza-associated hospitalisation estimates around the world and the importance of assessing the hospitalisation rates by age group. Because the estimation of influenza-associated hospitalisation rates is not straightforward and entails assumptions that are difficult to test, it is important to compare estimates from different modeling approaches, as we have done here. This information can be used to improve estimates of the burden of severe diseases, especially in lower- and middle-income countries, given their importance for vaccination program policies. Importantly, our estimate of 3.2 million hospitalisations fits the WHO estimates that annual influenza epidemics result in three to five million annual cases of severe illness (hospitalisations) per annum [1,5]. Finally, we found the highest influenza-associated hospitalisation rates were in children aged 0-4 and the elderly aged >65, groups often prioritized by prevention and control efforts.

## Additional material


Online Supplementary Document
