# Supplementary material for: Global and national influenza-associated hospitalisation rates: Estimates for 40 countries and administrative regions
Source: J Glob Health. 2023 Jan 27;13:04003. doi: 10.7189/jogh.13.04003 (PMC9879557; doi:10.7189/jogh.13.04003)
Supplement: Online Supplementary Document [file jogh-13-04003-s001.pdf]

ONLINE SUPPLEMENTARY DOCUMENT

Title: Global and national influenza-associated hospitalisation rates: estimates for 40 countries and administrative regions

Authors: Paget J, Staadegaard L, Wang X, Li Y, van Pomeren T, van Summeren J, Dückers M, Chaves SS, Johnson EK, Mahé C, Nair H, Viboud C, Spreeuwenberg P.

I. Literature reviews

English-speaking database search

Literature review search terms

1. hospitalization, hospital admission, committal, hospital episode
2. "influenza or "flu"
3. "excess" or "attributable" or "influenza-associated" or "influenza-related" or "influenza-attributed" or "influenza-attributable" or "associated with influenza" or "attributable to influenza" or "attributed to influenza" or "related to influenza" or "flu-associated" or "flu-related" or "associated with flu" or "attributable to flu" or "attributed to flu" or "related to flu."

1. Embase
   - Search period: until 12-03-2020
   - 1183 results
   - After excluding duplicates: 479 results
2. Pubmed, updated search
   - Search period: 11-02-2019 to 12-03-2020
   - 184 results
   - After excluding duplicates: 180
3. Pubmed, original search
   - Search period: until 11-02-2019
   - 3259
   - After excluding duplicates: 3247

Studies were excluded based on the following exclusion criteria:

1. The full-text article was not available;
2. Influenza-associated hospitalization rate was only estimated for a specific risk population, such as pregnant women or those with underlying medical conditions*;
3. The influenza-associated hospitalization rate only focused on the 2009 influenza pandemic;
5. The study investigators reported on a subset of complete data published elsewhere;
6. The collected data was from before 1995;
7. Less than 50 lab-confirmed cases (e.g. P&I or ARLI) were measured in a season/year.

Method

We performed a total of three searches in Pubmed and Embase, for studies reporting influenza-associated hospitalisations. Based on the original BIRD search from 2019, we updated our Pubmed search from 11-02-2019 to 12-03-2020. Additionally, we performed a new search in Embase, following the original search terms, but applied to Embase.

Results

Our searches resulted in a total of 4625 records. After removing duplicates, 3906 records remained for title and abstract screening by two reviewers. This resulted in 312 records that were assessed for full text. A total of 135 studies were included for data extraction.

Chinese-speaking database search

Method

We searched three Chinese-language databases, CNKI, Wanfang and Chongqing VIP for studies reporting flu-ALRI hospitalisations. The search strategy for the Chinese databases was adapted from the English search strategy. The same inclusion and exclusion criteria were applied to the Chinese-language studies.

Results

Our search resulted in 3015 records. After removing duplicates, 2630 records were screened by title and abstract, of which 24 records were screened by full-text. PRISMA flowchart is attached in **Figure 1**. A total of 7 studies were included in the review.

1. CNKI
   - （流感 AND 住院）OR（流感 AND 入院）
   - Publication period: 01/01/1995-31/12/2019
   - 476 results
2. Wanfang
   - （流感 AND 住院）OR（流感 AND 入院）
   - 学科分类：医药、卫生
   - Publication years: 1995-2019
   - 2313 results
3. Chongqing VIP
   - （流感 AND 住院）OR（流感 AND 入院）
   - Publication years: 1995-2019
   - 226 results

Translation: 流感=influenza; 住院=hospitalisation; 入院=hospital admission; 学科分类=subject; 医药=medicine; 卫生=health

Two teams of researchers independently screened all titles and abstracts for eligibility in the English language search (TvP and JvS) and the Chinese language search (YL and XW). Following this step, full-texts of the selected studies were independently screened for final inclusion by each researcher. Data from the selected studies were extracted into a standardized format, and were all double checked by a second independent researcher. Discrepancies were resolved by discussion with a third reviewer.

Hospitalization incidence rates were extracted as a rate per 100 000, and were adjusted accordingly if reported in another fashion. As both regional and national studies were considered, we collected the information to determine the geographical location of the study and the population from which the data were sampled, and information on the census population. Statistical methods that were performed to create incidence rates were extracted, and, when reported, corresponding 95% confidence intervals, ranges, standard errors, numerators and denominators as well.

Exclusion criteria

Studies were excluded if:

1. Full text was not available or we had no access,
2. The paper focused on the 2009 pandemic,
3. The study focused on a local hospital only,
4. The hospitalization rates were only reported for a particular subgroup such as pregnant women or those with comorbidities
5. The hospitalizations reported were but focused on ICU and/or ER admissions, or long-term care facilities,
6. The paper was in a language other than English or Chinese,
7. The data were derived from a vaccine effectiveness or efficacy study (e.g. a randomized control trial),
8. There were less than 50 confirmed cases were included in the study in a season/ year,
9. The rates were only reported in ranges (there were no point estimates). For duplicate results, we included the more detailed version of the study.

For the statistical analysis (see below), we excluded estimates or studies that did not fit a number of criteria:

- The data did not fit the age groups for which we had sufficient data i.e. children older than 5 years and adults up to the age of 59 years
- Studies did not provide 95% confidence intervals or standard errors as we wanted to provide confidence intervals to our estimates

II. Data extractions

**A**	Time stamp	Not applicable for manual data extraction
**B**	Author + year	First author of manuscript and year of publication
**C**	Country	Country on which study data is reported
**D**	National/regional	Does the study concern regional or national data?
**E**	Region (e.g. city & Province)	If regional study, specify region
**F**	Seasonal/Annual	Does the reported data concern seasonal or annual rates? Note: January – December of the same year is considered annual data, but also data collection running from July one year to June the following year.
**G**	Group	Group 1 = rate-based; Group 3=modelling-based
**H**	Population that was sampled (eligible)	Population from which sample was taken. For example, could be all hospitalized patients general, hospitalized SARI patients, hospitalized patients with general airway problems, all hospitalized ARI patients etc.
**I**	Envelope/ outcome measure	Clinical inclusion criteria/ category, such as ILI, ARI, SARI, P&I, R&C etc.
**J**	Envelope category	Did the envelope used sample a narrow subset of people, medium, or wide?<br>Narrow = lab-confirmed influenza, ICD code influenza and ICD code influenza (primary)<br>Medium = ICD code influenza (any (mention)), ARI, SARI, P&I, ILI etc<br>Wide = R&C, Respiratory, Circulatory, COPD, Asthma, Ischemic heart disease etc.
**K**	lab confirmed	Did the study use lab-confirmed data in the calculation of their rates? Answers either y(es)/ n(o)
**L**	ICD codes used	Did the study use ICD codes in the calculation of their rates? Answers either y(es)/ n(o)
**M**	Data source used	What is the data source that was used?
**N**	Statistical method	If applicable, what statistical method was used to create influenza hospitalization rates?
**O**	Start-year of data	Start year of data/ hospitalization rate
**P**	End_year of data	End year of data/ hospitalization rate
**Q**	Age_minimum	Lower range age category → in case of age categories as such: <1, then we calculate number of months into years. So, for example, Age category <1 would be 11 months – therefore noted as 0.92 (years)
**R**	Age_maximum	Upper range age category → in case of age categories as such: <1, then we calculate number of months into years. So, for example, Age category <1 would be 11 months – therefore noted as 0.92 (years)
**S**	Hospitalization rate per 100 000	Hospitalization rates reported, per 100 000. If paper reports rates per 1000 or 10 000, these need to be converted and entered as rates per 100 000.

**T**	Lower 95% CI	Lower range of 95% confidence interval, if reported
**U**	Higher 95% CI	Upper range of 95% confidence interval, if reported
**V**	Lower range	Lower range of rate, if rate is reported with corresponding ranges
**W**	Higher range	Higher range of rate, if rate is reported with corresponding ranges
**X**	SE	Standard error, if reported
**Y**	Census population/ catchment area	Catchment population in which study samples
**Z**	Census population, n=	Reported number of census population
**AA**	Census population year	Which year census population from column W is reported of
**AB**	Nominator (number of hospitalized influenza cases)	Nominator, in number of hospitalized influenza cases, if reported
**AC**	Denominator (size study population)	Denominator used, if reported
**AD**	Lower 95% CI	Lower range of 95% confidence interval, if reported
**AE**	Higher 95% CI	Upper range of 95% confidence interval, if reported
**AF**	SE	Standard error, if reported
**AG**	Vaccine effectiveness study	Are the rates extracted from a vaccine effectiveness study?
**AH**	Extracted by	Who extracted this data?
**AI**	Reviewed by	Who reviewed the data extraction?
**AO**	Comments	If applicable, any comments or notes on extraction

**AJ**	Sample population well defined	Is the sample population well defined?<br>Yes / No / Unclear
**AK**	Case definition well defined	Has the case definition been well defined?<br>Yes / No / Unclear
**AL**	Census population well defined	Has the census population been well defined?<br>Yes / No / Unclear
**AM**	Statistical methods reproducible	Are the statistical methods that were used to create the hospitalization rates reproducible? Yes / No / Unclear
**AN**	Rate calculations clear	Are the calculations performed to create the hospitalization rates clear and well described? Yes / No / Unclear

Abbreviations:

ARI= Acute Respiratory Infection
SARI = Severe Acute Respiratory Infection
P&I = Pneumonia and Influenza
R&C = Respiratory and Circulatory
ILI = Influenza Like Illness
COPD = Chronic Obstructive Pulmonary Disease
ICD = International Statistical Classification of Diseases and Related Health Problems (ICD), which is a medical classification list by the World Health Organization (WHO) that’s used to code medical diagnoses in hospitals.

ARI= Acute Respiratory Infection
SARI = Severe Acute Respiratory Infection
P&I = Pneumonia and Influenza
R&C = Respiratory and Circulatory
ILI = Influenza Like Illness
COPD = Chronic Obstructive Pulmonary Disease
ICD = International Statistical Classification of Diseases and Related Health Problems (ICD), which is a medical classification list by the World Health Organization (WHO) that’s used to code medical diagnoses in hospitals.

III. Definitions

Study types: Two types of studies were eligible for inclusion in the literature review: rate-based studies and time series regression-based studies. Rate-based studies were defined as studies that report age group specific hospitalization rates for influenza (generally based on laboratory confirmation or ICD coded diagnosis) (e.g. the multiplier method (4)). Time series regression-based studies were defined as studies that estimate excess hospitalizations using time series regression methods.

Papers with multiple outcomes: If a paper had hospitalisation estimates for multiple outcomes (e.g. 1) Pneumonia and Influenza and 2) Respiratory hospitalisations), we extracted all outcome estimates.

Envelopes: Considering the hospitalization outcome measures varied widely (5), we categorized the studies into three general envelopes based on the disease definition: 'narrow', 'medium', and 'wide. A 'narrow' envelope was defined as laboratory confirmed influenza and a clinical diagnosis of influenza (ICD code influenza in primary position). A 'medium' envelope was defined as any of the following disease outcomes without a (systematic) laboratory test confirmation: acute respiratory infection (ARI), influenza-like illness (ILI), acute lower respiratory tract infection (ARLI), severe acute respiratory infection (SARI), lower respiratory tract infection (LRTI), upper respiratory tract infection (URTI), pneumonia & influenza (P&I). A 'wide' envelope was defined as any of the following disease outcomes without a (systematic) laboratory test confirmation: circulatory and respiratory diseases, chronic obstructive pulmonary disease (COPD), stroke, critical illness, congestive heart failure, LRTI & pulmonary diseases.

Papers with multiple country estimates: If a paper had multiple country estimates (e.g. three countries), we extracted all estimates.

Season: In temperate countries a season was the winter period (e.g. rate from week 40 to week 20 in the Northern Hemisphere) and in tropical countries it was typically the year (rate over a 12-month period).

IV. Statistical multilevel meta-analysis model

The meta-analysis is done within a multilevel framework with binary outcomes. An advantage of this approach is that it is flexible enough to model the heterogeneous designs of the studies reported in the literature, and to separate the different influences this has on the outcomes. For a more elaborate discussion of these models we refer to the literature (see below)

$$Y_{i(j,k)} = \beta_0 + \sum_{r=1}^{5} \beta_r X + \mu_i + \mu_j + \mu_k + \epsilon_{i(j,k)}$$

I = individual measurement

J = study level

K = country (cross classified) level

$Y_{i(j,k)}$ = outcome measure, hospitalization rate

β_0 = intercept, modelled average hospitalization rate

β_rX = fixed effects for the five factors (r) , coding, (0/1) –(1/$N_{(categories)}$)

μ_i = between measurement variance

μ_j = between study variance

μ_k = between country variance

$\epsilon_{i(j,k)}$ = binomial error variance, constrained to 1

Notes:

(1) In this model the individual measurement level has two random components (error variance and between measurement variance)

(2) In this model the studies are not weighted, as is done in many meta-analysis studies. The reason is that the papers in the literature are so diverse in design and quality that no reasonable weights could be constructed.

Literature:

Fernández-Castilla B, Maes M, Declercq L, Jamshidi L, Beretvas S.N, Onghena P, Van den Noortgate W. A demonstration and evaluation of the use of cross-classified random-effects models for meta-analysis. Behavior Research Methods (2019) 51:1286-1304

Turner R.M, Omar R.Z, Yang M, Goldstein H, Thompson S.G. A multilevel model framework for meta-analysis of clinical trials with binary outcomes. Statistics in Medicine (2000) 19:3417-3432

V. Supplementary Table S1: Full list of included papers following the systematic review

Author	Title	Source	Location	WHO region	Included (Y/N)
Babakazo 2018	The national and provincial burden of medically attended influenza-associated influenza-like illness and severe acute respiratory illness in the Democratic Republic of Congo, 2013-2015	Influenza and Other Respiratory Viruses, 12(6), 695–705	Democratic Republic of Congo	African Region	Y
Dawa 2018	National burden of hospitalized and non-hospitalized influenza-associated severe acute respiratory illness in Kenya, 2012-2014	Influenza and Other Respiratory Viruses, 12(1), 30–37	Kenya	African Region	Y
Emukule 2014	The Burden of Influenza and RSV among Inpatients and Outpatients in Rural Western Kenya, 2009–2012	PLoS ONE, 9(8), e105543	Karemo division, Kenya	African Region	Y
Emukule 2019	Influenza-associated pneumonia hospitalizations in Uganda, 2013-2016	PLoS ONE, 14(7), e0219012	Wakiso district, Uganda	African Region	Y
Feikin 2012	The population-based burden of influenza-associated hospitalization in rural western Kenya	Bull World Health Organ, 90(), 256–263	Bondo district, Kenya	African Region	Y
Fuller 2013	Estimation of the National Disease Burden of Influenza-Associated Severe Acute Respiratory Illness in Kenya and Guatemala: A Novel Methodology	PLoS ONE, 8(2), e56882	Kenya	African Region	Y
McMorrow 2015	The Unrecognized Burden of Influenza in Young Kenyan Children, 2008-2012	PLOS ONE, 10(9), e0138272	Lwak, Kenya	African Region	Y
Ntiri 2016	Incidence of medically attended influenza among residents of Shai-Osudoku and Ningo-Prampram Districts, Ghana, May 2013 - April 2015	BMC Infectious Diseases, 16(1), 757	Shai-Osudoku & Ningo Pram-Pram (SONP) districts, Ghana	African Region	Y
Nyamusore 2018	The national burden of influenza-associated severe acute respiratory illness hospitalization in Rwanda, 2012-2014	Influenza and Other Respiratory Viruses, 12(1), 38–45	Rwanda	African Region	Y

Rabarison 2019	Burden and epidemiology of influenza- and respiratory syncytial virus-associated severe acute respiratory illness hospitalization in Madagascar, 2011-2016	Influenza and other Respiratory Viruses, 13(2), 138–147	Madagascar	African Region	Y
Theo 2018	The national burden of influenza-associated severe acute respiratory illness hospitalization in Zambia, 2011-2014	Influenza and other Respiratory Viruses, 12(1), 46–53	Zambia	African Region	Y
Abdel-Hady 2018	Estimating the burden of influenza-associated hospitalization and deaths in Oman (2012-2015)	Influenza and Other Respiratory Viruses, 12 (1), 146-152	Oman	Eastern Mediterranean Region	Y
Al-Awaidy 2015	The burden of influenza-associated hospitalizations in Oman, January 2008-June 2013	PLoS ONE, 10 (12), e0144186	Oman	Eastern Mediterranean Region	Y
Refaey 2016	Incidence of influenza virus-associated severe acute respiratory infection in Damanhour district, Egypt, 2013 2013	Eastern Mediterranean Health Journal, 22(7), 500	Damanhour district, Egypt	Eastern Mediterranean Region	Y
Ajayi-Obe 2008	Influenza A and respiratory syncytial virus hospital burden in young children in East London	Epidemiology and Infection, 136 (8), 1046–1058	East London, United Kingdom	European region	Y
Cromer 2014	The burden of influenza in England by age and clinical risk group: A statistical analysis to inform vaccine policy	Journal of Infection, 68(4), 363–371	England, United Kingdom	European region	Y
Fernandez 2019	Child hospital admissions associated with influenza virus infection in 6 Spanish cities (2014-2016)	Anales de Pediatria, 90(2), 86–93	Madrid, Basque Country, Barcelona & Malaga, Spain	European region	Y
Gefenaite 2018	Estimating burden of influenza-associated influenza-like illness and severe acute respiratory infection at public healthcare facilities in Romania during the 2011/12-2015/16 influenza seasons	Influenza and other Respiratory Viruses, 12(1), 183–192	Romania	European region	Y
Gil 2006	Hospitalization for pneumonia and influenza in the 50-64 year old population in Spain (1999-2002)	Human Vaccines , 2(4), 181–184	Spain	European region	N
Haas 2016	Burden of influenza in Germany: a retrospective claims database analysis for the influenza season 2012/2013	European Journal of Health Economics, 17(6), 669–679	Germany	European region	N

Hauge 2019	Burden of medically attended influenza in Norway 2008-2017	Influenza and Other Respiratory Viruses, 13(3), 240–247	Norway	European region	Y
Jacks 2012	Influenza-associated hospitalisations in Finland from 1996 to 2010: Unexpected age-specific burden during the influenza A(H1N1)pdm09 pandemic from 2009 to 2010	Eurosurveillance, 17(38), 2	Finland	European region	Y
Jansen 2007	Influenza- and respiratory syncytial virus-associated mortality and hospitalisations	European Respiratory Journal, 30(6), 1158–1166	Netherlands	European region	Y
Matias 2016	Modelling estimates of age-specific influenza-related hospitalisation and mortality in the United Kingdom	BMC Public Health, 16(1), 481	United Kingdom	European region	Y
Oliva 2018	Estimating the burden of seasonal influenza in Spain from surveillance of mild and severe influenza disease, 2010-2016	Influenza and other Respiratory Viruses, 12(1), 161–170	Spain	European region	Y
Pitman 2007	Assessing the burden of influenza and other respiratory infections in England and Wales	Journal of Infection, 54(6), 530–538	England & Wales, United Kingdom	European region	Y
Pivette 2020	Characteristics of hospitalizations with an influenza diagnosis, France, 2012-2013 to 2016-2017 influenza seasons	Influenza and other Respiratory Viruses, 14(3), 340–348	France	European region	Y
Rodrigues 2018	Excess pneumonia and influenza hospitalizations associated with influenza epidemics in Portugal from season 1998/1999 to 2014/2015	Influenza and other Respiratory Viruses, 12(1), 153–160	Portugal	European region	Y
Sakkou 2011	Impact of influenza infection on children's hospital admissions during two seasons in Athens, Greece	Vaccine, 29(6), 1167–1172	Athens, Greece	European region	N
San-Roman-Montero 2019	Inpatient hospital fatality related to coding (ICD-9-CM) of the influenza diagnosis in Spain (2009–2015)	BMC Infectious Diseases, 19(700),	Spain	European region	Y
Scuffham 2004	Estimating influenza-related hospital admissions in older people from GP consultation data	Vaccine, 22(21-22), 2853–2862	Switzerland	European region	N
Ampofo 2006	Epidemiology, complications, and cost of hospitalization in children with laboratory-confirmed influenza infection	Pediatrics, 118 (6), 2409-2417	Salt Lake City, Utah, United States	Region of the Americas	N

Ao 2019	Hospitalization and death among patients with influenza, Guatemala, 2008-2012	BMC Public Health, 19(suppl 3, 463)	Santa Rosa & Quetzaltenango, Guatemala	Region of the Americas	Y
Appiah 2015	Influenza activity - United States, 2014-15 season and composition of the 2015-16 influenza vaccine.	MMWR. Morbidity and mortality weekly report, 64(21), 583–90	United States	Region of the Americas	N
Azziz-Baumgartner 2012	Seasonality, timing, and climate drivers of influenza activity worldwide	Journal of Infectious Diseases, 206(6), 838–846	Argentina	Region of the Americas	Y
Bundy 2010	Burden of influenza-related hospitalizations among children with sickle cell disease	Pediatrics, 125(2), 234–243	California, New York, Florida & Maryland, United States	Region of the Americas	N
CDC 2008	Influenza Activity — United States and Worldwide, 2007–08 Season	MMWR. Morbidity and Mortality Weekly Report, 57(49), 1329-1332	United States	Region of the Americas	N
CDC 2011	Update: Influenza Activity - United States, October 2 - November 26, 2011	MMWR. Morbidity and Mortality Weekly Report, 60(48), 1646-1649	United States	Region of the Americas	N
CDC 2012	Update: Influenza Activity — United States, September 30–November 24, 2012	MMWR. Morbidity and Mortality Weekly Report, 61(48), 990-993	United States	Region of the Americas	Y
CDC 2013	Update: Influenza Activity — United States, September 29–December 7, 2013	MMWR. Morbidity and Mortality Weekly Report, 62(50), 1032-1036	United States	Region of the Americas	Y
CDC 2014	Update: Influenza Activity — United States, September 28–December 6, 2014	MMWR. Morbidity and Mortality Weekly Report, 63(50), 1189-1194	United States	Region of the Americas	N
CDC 2015	Update: Influenza Activity — United States, October 4–November 28, 2015	MMWR. Morbidity and Mortality Weekly Report, 63(50), 1342 - 1348	United States	Region of the Americas	Y
CDC 2016	Update: Influenza Activity — United States, October 2–December 17, 2016	MMWR. Morbidity and Mortality Weekly Report, 65(5051), 1439–1444	United States	Region of the Americas	Y

CDC 2017	Update: Influenza Activity — United States, October 1–November 25, 2017	MMWR. Morbidity and Mortality Weekly Report, 66(48), 1318–1326	United States	Region of the Americas	Y
CDC 2018	Influenza Activity — United States, September 30–December 1, 2018	MMWR. Morbidity and Mortality Weekly Report, 67(49), 1369–1371	United States	Region of the Americas	Y
CDC 2019	Update: Influenza Activity — United States, September 30, 2018–February 2, 2019	MMWR. Morbidity and Mortality Weekly Report, 68(6), 125 - 134	United States	Region of the Americas	Y
Chaves 2014	The Burden of Influenza Hospitalizations in Infants From 2003 to 2012, United States	Pediatric Infectious Disease Journal, 33(9), 912–919	United States	Region of the Americas	N
Chavez 2019	Estimation of influenza and respiratory syncytial virus hospitalizations using sentinel surveillance data—La Paz, Bolivia. 2012–2017	Influenza and other Respiratory Viruses, 13(5), 477–483	La Paz, Bolivia	Region of the Americas	Y
Cohen 2010	Trends for influenza and pneumonia hospitalization in the older population: Age, period, and cohort effects	Epidemiology and Infection, 138(8), 1135–1145	United States	Region of the Americas	Y
Cohen 2011	Influenza Vaccination in Young Children Reduces Influenza-Associated Hospitalizations in Older Adults, 2002-2006	Journal of the American Geriatrics Society, 59(2), 327–332	United States	Region of the Americas	N
Czaja 2020	State-level estimates of excess hospitalizations and deaths associated with influenza	Influenza and other Respiratory Viruses, 14(2), 111–121	Colorado, United States	Region of the Americas	N
Dao 2010	Adult Hospitalizations for Laboratory-Positive Influenza during the 2005–2006 through 2007–2008 Seasons in the United States	The Journal of Infectious Diseases, 202(6), 881–888	California,Colorado, Conneticut, Georgia, Maryland, Minnesota, New Mexico, New York, Oregon &Tennessee, United States	Region of the Americas	N
Dawood 2010	Burden of seasonal influenza hospitalization in children, United States, 2003 to 2008	Journal of Pediatrics, 157(5), 808–814	United States	Region of the Americas	N

Descalzo 2016	Estimating the burden of influenza-associated hospitalizations and deaths in Central America	Influenza and Other Respiratory Viruses, 10 (4), 340-345	Costa Rica, El Salvador, Guatemala, Honduras & Nicaragua	Region of the Americas	Y
Goldstein 2015	Estimating the hospitalization burden associated with influenza and respiratory syncytial virus in New York City, 2003-2011	Influenza and other Respiratory Viruses, 9(5), 225–233	New York City, United States	Region of the Americas	Y
Goldstein 2019	Hospitalizations associated with respiratory syncytial virus and influenza in children, including children diagnosed with asthma	Epidemiology, 30(6), 918–926	Arkansas, California, Colorado, Connecticut, Georgia, Hawaii, Iowa, Illinois, Indiana, Maryland, Minnesota, North Carolina, Nebraska, New Jersey, Nevada, New York, Ohio, Oregon, Tennessee, Texas, Virginia, Vermont, Washington, and Wisconsin, United States	Region of the Americas	N
Gounder 2014	Influenza hospitalizations among American Indian/Alaska Native people and in the United States general population	Open Forum Infectious Diseases, 1(1)	13 states, United States	Region of the Americas	Y
Grijalva 2006	Estimating influenza hospitalizations among children	Emerging Infectious Diseases, 12(1), 103–109	Davidson County	Region of the Americas	Y
Grijalva 2007	Estimating the undetected burden of influenza hospitalizations in children	Epidemiology and Infection, 135(6), 951–958	Davidson county (TN), Hamilton county (OH) &	Region of the Americas	Y

			Monroe county (NY), United States		
Grijalva 2010	The Population Impact of a Large School-Based Influenza Vaccination Campaign	PLoS ONE, 5(11), e15097	Knox county & surrounding counties, United States	Region of the Americas	N
Gruneir 2014	Influenza and seasonal patterns of hospital use by older adults in long-term care and community settings in Ontario, Canada	American Journal of Public Health, 104(2),	Ontario, Canada	Region of the Americas	N
Jules 2014	Age-Specific Influenza-Related Emergency Department Visits and Hospitalizations in 2010–2011 Compared With the Pandemic Year 2009–2010	Infectious Diseases in Clinical Practice, 22(5), 271–278	Davidson County, United States	Region of the Americas	Y
Kyeyagalire 2014	Hospitalizations associated with influenza and respiratory syncytial virus among patients attending a network of private hospitals in South Africa, 2007-2012	BMC Infectious Diseases, 14(1), 694	South Africa	Region of the Americas	Y
Libster 2010	Pediatric Hospitalizations Associated with 2009 Pandemic Influenza A (H1N1) in Argentina	New England Journal of Medicine, 362(1), 45–55	Buenos Aires, Argentina	Region of the Americas	N
Matias 2017	Estimates of hospitalization attributable to influenza and RSV in the US during 1997-2009, by age and risk status	BMC Public Health, 17(1), 271	United States	Region of the Americas	Y
Miller 2008	Influenza burden for children with asthma	Pediatrics, 121(1), 1–8	Nashville (TN), Rochester, (NY) & Cincinnati (OH), United States	Region of the Americas	N
Millman 2015	Improving accuracy of influenza-associated hospitalization rate estimates	Emerging Infectious Diseases, 21(9), 1595–1601	United States	Region of the Americas	Y
Ortiz 2014	Influenza pneumonia surveillance among hospitalized adults may underestimate the burden of severe influenza disease	PLoS ONE, 9(11), e113903	Arizona, California & Washington, United States	Region of the Americas	N

Poehling 2006	The Underrecognized Burden of Influenza in Young Children	New England Journal of Medicine, 355(1), 31–40	Nashville, Rochester (NY)& Cincinatti, United States	Region of the Americas	Y
Reed 2015	Estimating Influenza Disease Burden from Population-Based Surveillance Data in the United States	PLoS ONE 10(3): e0118369	United States	Region of the Americas	Y
Roberts 2006	Laboratory confirmed influenza associated hospitalizations among children in the metropolitan Toronto and Peel region by active surveillance, 2004-2005 - Canada.ca	Canada Communicable Disease Report, 32(18)	Peel & Toronto, Canada	Region of the Americas	N
Saborio 2014	Influenza-associated hospitalizations and deaths, Costa Rica, 2009-2012	Emerging Infectious Diseases, 20(5), 878–881	Costa Rica	Region of the Americas	Y
Schanzer 2006	Hospitalization attributable to influenza and other viral respiratory illnesses in Canadian children	Pediatric Infectious Disease Journal, 25(9), 795–800	Canada	Region of the Americas	N
Schanzer 2018	Burden of influenza, respiratory syncytial virus, and other respiratory viruses and the completeness of respiratory viral identification among respiratory inpatients, Canada, 2003-2014	Influenza and other Respiratory Viruses, 12(1), 113–121	Canada	Region of the Americas	Y
Schrag 2006	Multistate surveillance for laboratory-confirmed, influenza-associated hospitalizations in children 2003-2004	Pediatric Infectious Disease Journal, 25(5), 395–400	Denver, Georgia, Connecticut, Marland, Minnesota, New York, Oregon, Tennessee & Northern California area, United States	Region of the Americas	N
Sebastian 2008	Age-related trends in the timeliness and prediction of medical visits, hospitalizations and deaths due to pneumonia and influenza, British Columbia, Canada, 1998-2004	Vaccine, 26(10), 1397–1403	British Columbia, Canada	Region of the Americas	Y

Sotomayor 2018	Estimating the burden of influenza-associated hospitalizations and deaths in Chile during 2012-2014	Influenza and Other Respiratory Viruses, 12(1), 138–145	Chile	Region of the Americas	Y
Thompson 2004	Influenza-associated hospitalizations in the United States	Journal of the American Medical Association, 292(11), 1333–1340	United States	Region of the Americas	Y
Uscher-Pines 2013	Emergency Department Visits and Hospital Inpatient Stays for Seasonal and 2009 H1N1 Influenza, 2008-2009	HCUP Statistical Brief #147	United States	Region of the Americas	N
Widmer 2012	Rates of hospitalizations for respiratory syncytial virus, human metapneumovirus, and influenza virus in older adults	Journal of Infectious Diseases, 206(1), 56–62	Davidson County, United States	Region of the Americas	Y
Wong 2014	Influenza in Canada, 2012-2013 season	Canada Communicable Disease Report, 40(17), 346–354	British Columbia, Alberta, Saskatchewan, Manitoba, Ontario, Quebec, New Brunswick, Prince Edward Island, Nova Scotia, Newfoundland and Labrador, Yukon, Northwest Territories & Nunavut, Canada	Region of the Americas	Y
Zhou 2012	Hospitalizations Associated With Influenza and Respiratory Syncytial Virus in the United States, 1993–2008	Clinical Infectious Diseases, 54(10), 1427–1436	United States	Region of the Americas	N
Azziz-Baumgartner 2012	Incidence of influenza-like illness and severe acute respiratory infection during three influenza seasons in Bangladesh, 2008–2010	Bulletin of the World Health Organization, 90(1), 12–19	Bangladesh	South-East Asian Region	Y
Chadha 2013	Burden of Seasonal and Pandemic Influenza-Associated Hospitalization during and after 2009	PLoS ONE, 8(5)	Vadu, District Pune, India	South-East Asian Region	Y

	A(H1N1)pdm09 Pandemic in a Rural Community in India				
Hirve 2015	Incidence of influenza-associated hospitalization in rural communities in western and northern India, 2010-2012: A multi-site population-based study	Journal of Infection, 70(2), 160–170	Ballabgarhv & Vadu, India	South-East Asian Region	Y
Homaira 2016	Respiratory Viruses Associated Hospitalization among Children Aged <5 Years in Bangladesh: 2010-2014	PLOS ONE, 11(2), e0147982	Bangladesh	South-East Asian Region	Y
Simmerman 2009	Incidence, seasonality and mortality associated with influenza pneumonia in Thailand: 2005-2008	PLoS ONE, 4(11), e7776	Thailand	South-East Asian Region	N
Susilarini 2018	Estimated incidence of influenza-associated severe acute respiratory infections in Indonesia, 2013-2016	Influenza and Other Respiratory Viruses, 12(1), 81–87	Gunung Kidul District, Balikpapan district& Deli serdang district, Indonesia	South-East Asian Region	Y
Thapa 2019	The burden of influenza-associated respiratory hospitalizations in Bhutan, 2015-2016	Influenza and Other Respiratory Viruses, 13(1), 28–35	Bhutan	South-East Asian Region	Y
Anders 2015	Epidemiology and virology of acute respiratory infections during the first year of life: A birth cohort study in Vietnam	Pediatric Infectious Disease Journal, 34(4), 361–370	Ho Chi Minh City & Dong Thap, Vietnam	Western Pacific Region	N
Ang 2014	Influenza-associated hospitalizations, Singapore, 2004-2008 and 2010-2012	Emerging Infectious Diseases, 20(10), 1652–1660	Singapore	Western Pacific Region	Y
Ang 2017	Influenza-Associated Hospitalizations for Cardiovascular Diseases in the Tropics	American Journal of Epidemiology, 186(2), 202–209	Singapore	Western Pacific Region	N
Chiu 2002	Influenza-Related Hospitalizations among Children in Hong Kong	New England Journal of Medicine, 347(26), 2097–2103	Hong Kong	Western Pacific Region	N
Choi 2017	Disease burden of 2013-2014 seasonal influenza in adults in Korea	PLOS ONE, 12(3), e0172012	South Korea	Western Pacific Region	N

Coffin 2007	Incidence, complications, and risk factors for prolonged stay in children hospitalized with community-acquired influenza	Pediatrics, 119(4), 740–748	South Korea	Western Pacific Region	N
D'Onise 2008	The burden of influenza in healthy children in South Australia	Medical Journal of Australia, 188(9), 510–513	South Australia, Australia	Western Pacific Region	Y
Huang 2007	Influenza surveillance in New Zealand in 2005	The New Zealand Medicine Journal, 120(1256),	New Zealand	Western Pacific Region	Y
Huang 2008	Influenza surveillance and immunisation in New Zealand, 1997-2006	Influenza and other Respiratory Viruses, 2(4), 139–145	New Zealand	Western Pacific Region	Y
Ieng 2018	National burden of influenza-associated hospitalizations in Cambodia, 2015 and 2016	Western Pacific surveillance and response journal : WPSAR, 9(5), 44–52	Svay Rieng, Siem Reap & Kampong Cham, Cambodia	Western Pacific Region	Y
Khieu 2015	Estimating the contribution of influenza to hospitalisations in New Zealand from 1994 to 2008	Vaccine, 33(33), 4087–4092	New Zealand	Western Pacific Region	Y
Kim 2011	Trends in Pneumonia and influenza-associated hospitalizations in South Korea, 2002-2005	Journal of Health, Population and Nutrition, 29(6), 574–582	South Korea	Western Pacific Region	Y
Kohlmaier 2020	A severe influenza season in Austria and its impact on the paediatric population: Mortality and hospital admission rates, november 2017 - March 2018	BMC Public Health, 20(1)	Austria	Western Pacific Region	N
Li 2006	Influenza-related deaths and hospitalizations in Hong Kong: A subtropical area	Public Health, 120(6), 517–524	Hong Kong	Western Pacific Region	N
Nelson 2007	Assessing disease burden of respiratory disorders in Hong Kong children with hospital discharge data and linked laboratory data	Hong Kong Medical Journal, 13(2), 114–121	Hong Kong	Western Pacific Region	Y
Newall 2008a	Influenza-related disease: The cost to the Australian healthcare system	Vaccine, 26(), 6818–6823	Australia	Western Pacific Region	Y
Newall 2008b	Influenza-related hospitalisation and death in Australians aged 50 years and older	Vaccine, 26(17), 2135–2141	Australia	Western Pacific Region	Y

Ng 2019	Estimates of influenza-associated hospitalisations in tropical Singapore, 2010-2017: Higher burden estimated in more recent years	Influenza and other Respiratory Viruses, 13(6), 574–581	Singapore	Western Pacific Region	Y
Sheu 2016	Comparison of age-specific hospitalization during pandemic and seasonal influenza periods from 2009 to 2012 in Taiwan: A nationwide population-based study	BMC Infectious Diseases, 16(1), 88	Taiwan	Western Pacific Region	N
Stewart 2018	Using a hospital admission survey to estimate the burden of influenza-associated severe acute respiratory infection in one province of Cambodia—methods used and lessons learned	Influenza and other Respiratory Viruses, 12(1), 104–112	Svay Rieng Province, Cambodia	Western Pacific Region	Y
Wong 2006	Influenza-associated hospitalization in a subtropical city	PLoS Medicine, 3(4), 485–492	Hong Kong	Western Pacific Region	Y
Wong 2009	Influenza-associated hospitalisation	Hong Kong Med J, 15(), s35–s37	Hong Kong	Western Pacific Region	Y
Wu 2017	A joint analysis of influenza-associated hospitalizations and mortality in Hong Kong, 1998-2013	Scientific Reports, 7(1), 929	Hong Kong	Western Pacific Region	Y
Yang 2012	Excess mortality associated with the 2009 pandemic of influenza A(H1N1) in Hong Kong	Epidemiology and Infection, 140(9), 1542–1550	Hong Kong	Western Pacific Region	Y
Yang 2019	Comparison of influenza disease burden in older populations of Hong Kong and Brisbane: The impact of influenza and pneumococcal vaccination	BMC Infectious Diseases, 19(1)	Brisbane, Australia	Western Pacific Region	Y
Yap 2004	Excess hospital admissions for pneumonia, chronic obstructive pulmonary disease, and heart failure during influenza seasons in Hong Kong	Journal of Medical Virology, 73(4), 617–623	Hong Kong	Western Pacific Region	Y
Yoshihara 2019	Influenza B associated paediatric acute respiratory infection hospitalization in central vietnam	Influenza and other Respiratory Viruses, 13(3), 248–261	Nha Trang city, Vietnam	Western Pacific Region	Y
Yu 2014	The substantial hospitalization burden of influenza in central China: Surveillance for severe, acute	Influenza and other Respiratory Viruses, 8(1), 53–65	Two districts of Jingzhou City, China	Western Pacific Region	Y

	respiratory infection, and influenza viruses, 2010-2012				
Yu 2019	Influenza-associated Hospitalization in Children Younger Than 5 Years of Age in Suzhou, China, 2011-2016	Pediatric Infectious Disease Journal, 38(5), 445–452	Suzhou, China	Western Pacific Region	Y
Zhang 2017	Pneumonia and influenza hospitalizations among children under 5 years of age in Suzhou, China, 2005–2011	Influenza and other Respiratory Viruses, 11(1), 15–22	Suzhou, China	Western Pacific Region	Y
Zhang 2018	Hospitalizations for influenza-associatesevere acute respiratory infection, Beijing, China, 2014–2016	Emerging Infectious Diseases, 24(11), 2098–2102	Beijing, China	Western Pacific Region	Y
冯录召 2014	Technical guidelines for the application of seasonal influenza vaccine in China (2014-2015)	Chinese journal of Epidemiology,2014,35(12), 1295-1319	Wuxi, China	Western Pacific Region	N
张奕 2017	Estimating the burden of influenza-associated hospitalization for cases of severe acute respiratory infection, Beijing, 2015	Chinese Journal of Preventative medicine, 51 (12)	Beijing, China	Western Pacific Region	Y
张婉青 2019	Hospitalization rates for influenza-associated severe acute respiratory illness in children younger than five years old in Suzhou of China, 2016-2018	Chinese Journal of Preventive Medicine 2019,53 (10): 1056-1059	Suzhou, China	Western Pacific Region	Y
朱秋丽 2011	A retrospective study of influenza epidemiology and disease burden of hospitalized children in Suzhou	Doctoral thesis (Fudan University)	Suzhou, China	Western Pacific Region	Y
赵小娟 2019	2014-2016 Severe Acute Respiratory Infections in Huairou District, Beijing: Analysis of influenza virus infection and hospitalization rate	Pract Prev Me, 2019, 26(9), 1131-1134	Beijing, China	Western Pacific Region	Y
赵小娟 2018	Study on the influenza infection rate and incidence rate during the 2017-2018 epidemic season in Beijing	国际病毒学杂志 (Translation: international journal of virology) August 2018， Vol 25， No 4: 281-3	Beijing, China	Western Pacific Region	Y

郑建东 2015	Estimation of hospitalization rate of laboratory confirmed influenza cases in Jingzhou city, Hubei province, 2010-2012	Chinese Journal of Epidemiology, 2015, 36(3), 222-227	Jingzhou, China	Western Pacific Region	Y

VI. Supplementary Tables

Supplementary Table S2. Multilevel model outcomes

Panel A: Fixed effects: independent factors

Independent factors	All ages		Children aged 0-4		Elderly, aged 65+ years	
	Estimate	p-value	Estimate	p-value	Estimate	p-value
Intercept	-7.8206	-	-6.5861		-7.2700	
Age correction 1			0.1372	0.9097	1.0167	0.2561
Age correction 2			**-1.2085**	**0.0009**	1.5547	0.1547
Age correction 3			**-1.0770**	**0.0024**	-	-
**Factor 1:** Rate-based (vs. time series regression-based)	**1.0606**	**0.0236**	**1.5355**	**0.0002**	-0.1138	0.7931
**Factor 2:** Measurement outcome-medium	-0.2912	0.5602	**-1.8295**	**0.0323**	**-1.7437**	**0.0033**
**Factor 2:** Measurement outcome-wide	0.4694	0.3554	-1.1136	0.2009	-0.9971	0.1548
**Factor 3:** Laboratory test – Yes (vs. No)	**-1.7017**	**0.0008**	**-3.3495**	**0.0001**	**-3.2974**	**0.0000**
**Factor 4:** National (vs. subnational)	-0.5045	0.1243	-0.3762	0.2167	-0.2939	0.3466
**Factor 5:** One season (vs. Multiple seasons)	**0.5605**	**0.0123**	0.1173	0.4568	-0.2188	0.3977

Panel B: Random effects: three levels in the model

Levels	All ages		Children aged 0-4		Elderly, aged 65+ years	
	Variance	Standard error	Variance	Standard error	Variance	Standard error
Measurement outcome (level 2)	0.42735	0.04339	0.43473	0.04639	0.43648	0.056619
Study (level 3)	1.01310	0.33884	0.63478	0.21167	0.59307	0.23953
Country (level 4)	0.63144	0,40552	0.50404	0.25517	0.62918	0.29999

Supplementary Table S3. Pooled influenza-related hospitalization rates by age and country

WHO region and country	All ages		Children age 0-4		Elderly aged 65+	
	Average hospitalization rate per 100,000	95% Confidence Interval	Average hospitalization rate per 100,000	95% Confidence Interval	Average hospitalization rate per 100,000	95% Confidence Interval
**AFRO region**						
D.R. Congo	47.2	20.5-108.8	176.8	78.3-398.6	108.0	42.9-271.2
Ghana	36.1	15.7-83.1	123.6	57.4-265.7	67.4	26.9-168.7
Kenya	51.9	16.2-165.7	149.9	46.8-479.2	28.8	11.7-70.8
Madagascar	49.4	19.6-124.2	140.3	58.6-335.2	96.7	38.5-242.6
Rwanda	42.7	18.6-98.2	-	-	80.9	32.3-202.5
South Africa	39.4	16.4-94.3	-	-	-	
Uganda	37.7	15.3-93.3	80.6	33.7-192.7	68.1	24.5-189.2
Zambia	43.8	18.8-102.0	86.3	48.6-153.3	43.8	18.6-103.2
**EMRO region**						
Egypt	-	-	-	-	75.2	29.8-189.8
Oman	18.6	6.1-56.6	83.9	30.3-232.6	49.0	14.5-165.0
**EURO region**						
Finland	30.9	13.5-70.2	99.0	47.0-208.4	91.3	36.4-228.9
France	25.0	10.4-60.3	-	-	-	-
Netherlands	-	-	-	-	34.8	13.3-91.2
Norway	30.0	12.4-72.6	-	-	-	-
Portugal	37.4	15.0-93.2	-	-	49.2	17.8-136.0
Romania	18.6	7.5-45.8	-	-	-	-
Spain	24.3	8.6-68.4	81.4	28.6-232.0	64.2	20.8-197.5
United Kingdom	92.4	31.4-271.9	191.7	84.0-436.8	112.7	41.0-309.8
**PAHO region**						
Argentina	38.6	15.6-95.7	-	-	-	-
Bolivia	30.9	12.9-74.5	144.4	61.7-337.5	86.6	30.1-248.8
Canada	33.0	11.5-94.9	-	-	36.6	12.3-108.7
Chile	-	-	60.1	25.6-141.1	74.1	26.6-206.1
Costa Rica	52.8	22.8-121.9	59.9	18.2-197.2	75.1	20.0-281.1
El Salvador	-	-	210.3	65.7-671.4	91.5	24.4-342.7
Guatemala	48.9	19.3-123.4	76.4	23.1-252.9	46.1	11.8-179.1

Honduras	-	-	86.7	27.1-276.7	36.2	9.7-135.2
Nicaragua	-	-	221.6	69.2-707.4	74.3	19.8-278.0
United States	92.6	23.5-364.7	94.1	28.5-309.9	433.4	112.6-1653.2
**SEARO region**						
Bangladesh	-	-	216.3	78.6-594.3	-	-
Bhutan	71.3	28.4-179.4	207.0	87.0-491.5	-	-
India	122.1	41.5-358.5	-	-	162.3	50.1-525.0
Indonesia	33.2	13.5-81.4	120.1	50.8-283.6	-	-
**WPRO region**						
Australia	28.4	9.9-81.7	191.0	87.4-417.0	43.0	13.5-136.7
Cambodia	27.3	9.3-79.9	112.5	49.1-257.9	34.2	10.7-109.2
China - Mainland	73.0	24.9-214.1	364.0	110.5-1191.7	161.7	43.3-602.5
China-Hong Kong	35.7	11.8-107.5	243.1	93.7-629.3	78.3	23.6-260.1
New Zealand	11.7	3.8-36.3	-	-	-	-
Singapore	45.1	15.6-130.7	166.4	72.6-380.9	104.9	38.1-288.6
South Korea	86.4	34.6-216.0	338.0	144.1-791.1	-	-
Vietnam	-	-	216.3	92.6-504.7	-	-
